# Real-world treatment patterns of rheumatoid arthritis in Brazil: analysis of DATASUS national administrative claims data for pharmacoepidemiology studies (2010–2020)

**DOI:** 10.1038/s41598-023-44389-9

**Published:** 2023-10-18

**Authors:** Marina G. Birck, Rafaela Ferreira, M. Curi, Whitney S. Krueger, Guilherme S. Julian, Alexander Liede

**Affiliations:** 1 IQVIA Brasil, São Paulo, Brazil; 2 AbbVie, São Paulo, Brazil; 3 AbbVie Inc., North Chicago, IL USA; 4 Global Epidemiology, AbbVie, 14 Riverwalk, Citywest Business Campus, Dublin 24, D24 XN32 Ireland

**Keywords:** Epidemiology, Databases, Rheumatoid arthritis

## Abstract

Our study assessed DATASUS as a potential source for pharmacoepidemiologic studies in rheumatoid arthritis (RA) in the Brazilian population focusing on treatment patterns and determinants of initiating or switching to a novel therapy. This was a descriptive database study of RA patients with at least one claim of RA and ≥ 2 claims of disease-modifying anti-rheumatic drug (DMARD); conventional synthetic (cs), biologic (b) or targeted synthetic (ts) DMARD with more than 6 months of follow-up from 01-Jan-2010 to 31-Dec-2020. Analyses were stratified for SUS-exclusive and SUS+ private user cohorts. We identified 250,251 patients with RA in DATASUS: mean age of 58.4 years, majority female (83%) and white (58%). 62% were SUS-exclusive and 38% SUS+ private. Most common bDMARDs were adalimumab and etanercept. Age (adjusted odds ratio 1.78 [50+]; 95% CI 1.57–2.01), SUS exclusive status (0.53; 0.47–0.59), distance to clinic [160+ km] (0.57; 0.45–0.72), and pre-index csDMARD claims (1.23; 1.08–1.41) were independent predictors of initiating a novel oral tsDMARD. Switching from bDMARD to tsDMARD, associations were similar, except for the direction of associations for SUS exclusive status (adjusted hazard ratio 1.10; 1.03–1.18), distance to clinic (1.18; 1.03–1.35), and number of previous bDMARD (0.15; 0.14–0.16). DATASUS is a source suitable for treatment-related analyses in RA reflecting the public health system in Brazil.

## Introduction

Real-world evidence and insights inform on multiple aspects of the patient experience and disease journey, capturing patient characteristics, treatment selection, persistence and switching patterns, and safety-related outcomes associated with drug exposure^1–4^. Understanding drug utilization patterns in the real world is a vital step in building the benefit-risk profile of medicinal products, which ultimately inform recommendations for prescribing interventions to patients^5–7^.

Pharmacoepidemiology and drug utilization research are related disciplines and share the need for reliable sources of real-world data^7,8^. In addition to informing on the economics and health resource utilization, administrative insurance claims databases are often used in pharmacoepidemiology and pharmacovigilance research, such as with the United States Food and Drug Administration (FDA) Sentinel initiative^9,10^. In Brazil, the large national administrative claims database that represents the public healthcare system (SUS, *Sistema Único de Saúde*) is called DATASUS. Established by the Ministry of Health in 1990, DATASUS contains information and statistics from all municipalities in Brazil and is publicly available through online access to researchers (http://datasus.saude.gov.br/), integrating ambulatory (out-patient)^11^ and hospital (in-patient)^12^ databases and capturing high cost or complex drug dispensation and procedures that are covered by SUS^13^. Although it is the universal healthcare system in Brazil, more than 75% (~ 150 million) of the population are exclusively dependent on SUS, whereas the remainder of Brazil’s population have supplementary private health insurance plans, and therefore may access SUS episodically.^14,15^

Rheumatoid arthritis (RA) is a chronic, immune-mediated inflammatory disease affecting with a global prevalence of 460 per 100,000 (0.46%; 95% prediction interval [0.06–1.27])^16^, and a prevalence rate of 200–1000 per 100,000 in Brazil^17,18^. DATASUS has been used for studies examining patient characteristics and patterns of treatment in other chronic conditions such as multiple sclerosis^19^, spinal muscular atrophy^20^, breast cancer^21^, and age-related macular degeneration (AMD)^22^.

This study aimed to evaluate DATASUS as a suitable data source for the conduct of pharmacoepidemiologic studies in the RA population of Brazil. Among the various treatment options are available within SUS, we focused on treatment patterns associated with disease-modifying antirheumatic drug (DMARDs), immunosuppressive and immunomodulatory interventions classified as conventional synthetic (cs), biologic (b) or targeted synthetic (ts) DMARDs^15,18^.

## Methods

### Study design and database

This was a descriptive, retrospective claims database study using the DATASUS database. The study identified patients with RA who sought care within SUS from January 1, 2010, to December 31, 2020.

The administrative claims data in DATASUS are presented as procedure codes from billing records and include demographic information, all procedures (in-patient and out-patient), costs, and additional information^23^. In-patient (SIH [*Sistema de Informações Hospitalares*])^24^ and out-patient (SIA [*Sistema de Informações Ambulatoriais*])^11^ data exist separately, and were connected at the patient level using multiple steps with different combinations of individual-level information (date of birth, sex, city, and postal code) for a probabilistic linkage approach. This Brazilian Healthcare Record Linkage (BRHC-RLK) methodology has been used in previous studies to enable more comprehensive capture of each patient’s health record and thus a more complete evaluation of their journey across the SUS system^25^. The method relies on multiple steps with different combinations of patient information from both databases, making it possible to identify or link patient data in both systems while maintaining the de-identified nature of the database.

Patient-level data within DATASUS are anonymized and encrypted before being made available to researchers. DATASUS is publicly available, and does not require further approval from ethics committees, according to the Brazilian ethics Resolution n° 510/2016.

### Study population

The study population included patients with at least one claim of RA (per International Classification of Disease, 10th edition [ICD-10] codes: M05.0, M05.3, M05.8, M06.0, M06.8, or M08.0) and ≥ 2 claims for disease-modifying antirheumatic drugs (DMARDs) ≥ 1 month apart in the 2010–2020 study period. This study examined a treated population with index date being the first DMARD claim and follow up until end of the study period (31-Dec-2020) or last information available. Detailed DMARD definitions are provided in Supplementary Table S1.

In order to capture the initial treatment and address potential for misclassification common with claims data, patients with any claim of DMARD without an RA ICD-10 code 12 months prior to the index date were excluded. The index date was defined as the date of the first RA ICD-10 and DMARD prescription in the public health system during the study period. Patients with RA with less than 6 months of follow-up were excluded, in an attempt to reduce individuals with a false diagnosis or lack of follow-up in the database.

As SUS is a universal coverage healthcare system, patients who have supplementary private health insurance plans may also obtain medications (such as high-cost medications) covered by SUS without out-of-pocket expense. This is commonly observed in other therapeutic areas^26^. For this reason, we stratified results across the following cohorts: Cohort 1 being the full study population, Cohort 2 SUS-exclusive (i.e., dependent on SUS for all healthcare related encounters, procedures, and treatments), and Cohort 3 representing SUS + private patients (i.e., only dependent on SUS for prescription drug coverage)^26^.

### Measurements of DMARD treatment

DMARD treatments measured using procedure codes (see Supplementary Table S1) were grouped into the following categories: csDMARD for conventional synthetics and/or immunosuppressants (ciclosporin, cyclophosphamide, chloroquine, hydroxychloroquine, leflunomide, methotrexate, azathioprine, and sulfasalazine), bDMARD for biologics (adalimumab, abatacept, etanercept, infliximab, rituximab, tocilizumab, golimumab, certolizumab), and tsDMARD for a target synthetic, oral therapy [Janus kinase (JAK) inhibitor, tofacitinib].

Treatment patterns were evaluated by specific drug (independently of monotherapy or in combination) as dispensed for RA treatment and the sequence of the available treatments in SUS, by lines of therapy (LOT), time of each drug, previous and subsequent DMARD treatments in SUS. The first treatment was the first therapy from the inclusion per RA ICD-10 code. LOT was defined as at least three claims (dispensation) of the same drug (b/tsDMARD) in sequence. A new sequence of at least three claims (dispensation) of the same drug in sequence was considered a new treatment-line. Thus, the treatment switch was identified as at least three claims of different drug(s) than the previous one, not included as the definition of drugs used in combination. Gaps were allowed, regardless of time, and did not define a new LOT. First line (LOT1) refers to initial treatment, first b/tsDMARD claim of RA during the study period. Second-line (LOT2) refers to the second b/tsDMARD used for RA treatment, when the first b/tsDMARD was stopped. Third line (LOT3) refers to the third b/tsDMARD, when the previous b/tsDMARD was terminated. csDMARDs used prior to b/tsDMARD were also assessed. Treatment switch was defined as at least three claims of different drug(s) than the previous one (new LOT), and that was not part of drugs used in combination.

### Statistical analyses

Derived variables included age and distance from clinic. Age was defined as the age at the first claim of an ICD-10 code for RA in the database. Distance was calculated as the Euclidean distance (km) between two postal codes: patient’s residence and the health care facility or tomography or antiangiogenic treatment institution, as applicable. Treatment switch, discontinuations, and end of follow up were main outcomes or censoring events of interest, also relevant in defining LOT and creation of Sankey diagrams.

Continuous variables (e.g., age) are summarized by central tendency (means, medians) and spread (variance, range); and for categorical variables (e.g., sex) by absolute number and percentage. Stratifications and/or sensitivity analysis were done to evaluate differences in gender, age groups, patients’ region of residence, drug use, treatment line, and others.

Stratified analyses for prevalent and new users were prespecified, and SUS-exclusive and SUS+ private cohorts. Prevalent users were patients with RA currently receiving bDMARD treatment, and new users were patients with RA initiating a new bDMARD treatment (i.e., their first prescription). For describing use and sequential patterns of RA bDMARD treatments, patients were stratified by treatment type, LOT specific drug, and SUS-exclusive status.

In multivariable logistic regression analyses, age, SUS-exclusive status, distance to clinic (160+ km), and pre-index cs/imsDMARD and others were independent predictors to evaluate the initiate therapy (LOT1) with b/tsDMARD (JAKi). Multivariable analyses were performed using Cox regression models that evaluated predictors to time to switch therapy to tsDMARD (JAKi) compared to bDMARD (LOT2+) applying the same independent predictors of multivariable logistic regression analyses (age, SUS-exclusive status, distance to clinic, pre-index cs/imsDMARD, others), plus number of prior bDMARD used. Sankey diagrams were used for treatment pattern visualizations. Sankey diagrams quantitatively illustrate treatment sequencing (and/or time on treatment) and allow stratification by subpopulations of interest with censoring by treatment switch, discontinuation, or end of follow up. Kaplan Meier survival analyses and plots were generated for time to switch from LOT1 to LOT2, among those treated with b/tsDMARD, analyzed by type of drug, and by SUS-exclusive status.

The visual representation of time-to-event of switch from LOT1 to LOT2 among patients receiving b/tsDMARD therapies was represented in Kaplan–Meier curves. The last information available of the patient or the end of the study period was considered censored for patients who did not switch from LOT1 to LOT2.

All analyses were performed using python version 3.6.9 and statistical significance was set at p < 0.05.

## Results

### Cohort creation and patient demographics

**Figure 1:**
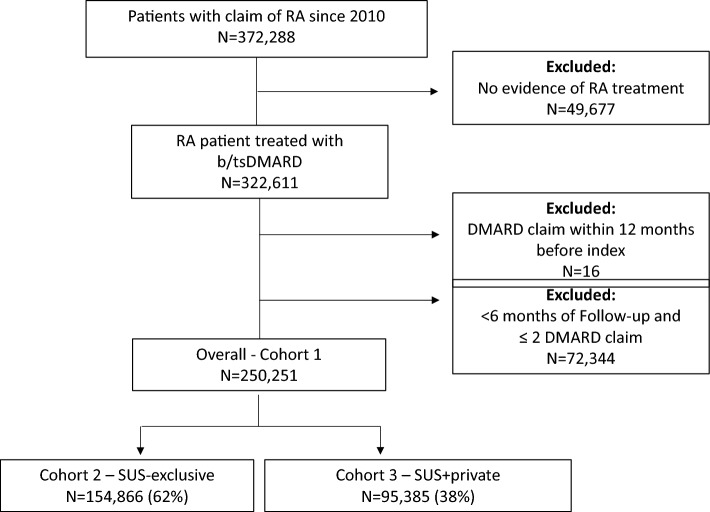
Study population of b/tsDMARD-treated rheumatoid arthritis patients in DATASUS with cohorts defined by their dependency on Brazil´s public health system (Sistema Único de Saúde), either exclusively (SUS_exclusive) or in combination with private insurance coverage (SUS+ private) (STROBE diagram). *RA* rheumatoid arthritis, *DMARD* disease modifying antirheumatic drug, *N* total number of patients, *SUS* Sistema Único de Saúde, *SUS-exclusive* comprehensive public health system for the entire population, *SUS+ private* refers to individuals on SUS only for prescription drug coverage.

Of the total 250,251 patients with RA included in the study (Cohort 1), 154,866 (61.9%) patients were part of the SUS-exclusive Cohort 2, and 95,385 (38.1%) patients were considered SUS+ private Cohort 3. Figure 1 depicts the STROBE diagram for study cohort creation. Mean ages (58.4, 58.1, and 58.7) and medium ages (58.0, 57.0, and 58.0) were comparable across cohorts (58.4, 58.1, and 58.7 for Cohort 1, 2, and 3, respectively) (Table 1). Across cohorts, most were female (82.8%, 84.5%, and 80% for Cohort 1, 2, and 3 respectively) and white (57.7%, 58.6%, and 54.2% for Cohort 1, 2, and 3 respectively). Over half of patients (52.9%) were from the southeast region of Brazil, representing 51.5% in the SUS-exclusive and 55.2% in the SUS+ private cohorts. Median (inter-quartile range) follow-up was 3.4 (1.6–6.3) years overall, 3.3 (1.6–6) years for Cohort 2 and 3.6 (1.7–6.8) years for Cohort 3.

**Table 1:** Characteristics of patients with rheumatoid arthritis (overall), cohort 2 [SUS-exclusive—individuals with full public health coverage (SUS)] and cohort 3 (SUS+ private—individuals dependent on SUS only for prescription drug coverage).

	SUS-exclusive (Cohort 2), N = 154,866	SUS+ private (Cohort 3), N = 95,385	Overall (Cohort 1), N = 205,251
Age*, years, mean (SD)	58.1 (14.9)	58.7 (14.9)	58.4 (15.3)
Gender, N (%)			
Female	130,836 (84.5)	76,342 (80.0)	207,178 (82.8)
Male	24,030 (15.5)	19,043 (20.0)	43,073 (17.2)
Ethnicity, N (%)			
White	83,828 (58.6)	20,464 (54.2)	104,292 (57.7)
Mixed	43,981 (30.7)	13,477 (35.7)	57,458 (31.8)
Black	7058 (4.9)	1210 (3.2)	8268 (4.6)
Indigenous	84 (0.1)	15 (0)	99 (0.1)
Asian	8096 (5.7)	2604 (6.9)	10,700 (5.9)
Missing	11,819 (7.6)	57,615 (60.4)	69,434 (27.7)
Residence, N (%)			
Southeast	78,399 (51.5)	51,355 (55.2)	129,754 (52.9)
South	34,673 (22.8)	18,911 (20.3)	53,584 (21.9)
Northeast	26,586 (17.5)	15,416 (16.6)	42,002 (17.1)
Central West	6413 (4.2)	4760 (5.1)	1,1173 (4.6)
North	6039 (4.0)	2661 (2.9)	8700 (3.5)
Missing	2756 (1.8)	2282 (2.4)	5038 (2.0)
Follow-up time**, years			
Median (IQR)	3.3 (1.6–6)	3.6 (1.7–6.8)	3.4 (1.6–6.3)
b/tsDMARD at index date			
Adalimumab	25,689 (38)	21,121(42)	46,810 (40)
Certolizumab	4084 (6)	2324 (5)	6408 (5)
Etanercept	17,897 (26)	14,213 (28)	32,110 (27)
Golimumab	4940 (7)	3687 (7)	8627 (7)
Infliximab	7039 (10)	4567 (9)	11,606 (10)
Abatacept	2167 (3)	1081 (2)	3248 (3)
Rituximab	1875 (3)	956 (2)	2831 (2)
Tocilizumab	2268 (3)	1208 (2)	3476 (3)
Tofacitinib	1630 (2)	1382 (2)	3012 (3)

The index date was defined as the date of the first RA ICD-10 and DMARD prescription in the public health system during the study period.

Residence: regional localization of primary home address, *IQR* interquartile range, *N* number of patients, *SD* standard deviation, *SUS* Sistema Único de Saúde, SUS-exclusive: comprehensive public health system for the entire population, SUS+ private: only relying on SUS for medication.

*Age at index date (treatment initiation).

**Since index date.

### Treatment patterns and trends of RA drug use

**Figure 2:**
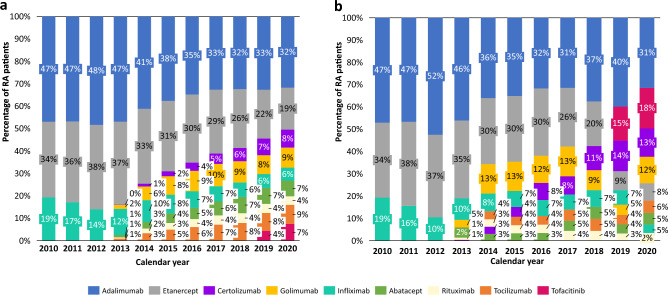
Prevalent users (**a**) *and new users* (**b**) of b/tsDMARD treatment among patients with rheumatoid arthritis in DATASUS per year. Prevalent users: patients with RA currently receiving b/tsDMARD treatment, percentage shown by b/tsDMARD type by year. New users: patients with RA initiating a new b/tsDMARD treatment (i.e., their first prescription). *b/tsDMARD* biologic/targeted synthetic DMARD. tsDMARD limited to tofacitinib at time of analysis.

In 2010, the most common bDMARD among prevalent users were adalimumab (47%), etanercept (34%) and infliximab (19%) (Fig. 2). In 2020, the most common were adalimumab (32%), etanercept (19%) and golimumab (9%). The most common b/tsDMARDs among new users in 2020 were adalimumab (31%), tofacitinib (18%) and certolizumab (13%) (Fig. 2). The predominance of adalimumab and etanercept declined steadily in both prevalent and new user groups from 2013 onwards due to the introduction and emerging use of other bDMARDs (rituximab, tocilizumab, certolizumab, and golilumab, among a few others); nonetheless, for prevalence group, adalimumab and etanercept continued to be the most common drug of choice by the end of the study period in 2020.

### Predictors for initiating or switching with tsDMARD

Table 2 shows the results of the logistic and Cox regression evaluating determinants of initiating or switching to a new tsDMARD, respectively.

**Table 2:** Regression models examining predictors of rheumatoid arthritis treatment initiation of tsDMARD (JAKi) or switch to tsDMARD from bDMARD.

	Treatment initiation with JAKi vs. bDMARDs (logistic regression) Odds ratio (95%CI)		Treatment switch to JAKi from bDMARD (Cox proportional hazards) Hazard ratio (95%CI)	
Independent variables	Univariate	Multivariate	Univariate	Multivariate
Female	1.10 (0.95; 1.28)	1.09 (0.94; 1.27)	0.98 (0.88; 1.08)	0.99 (0.90; 1.10)
> 50 years at first RA ICD claim	1.73 (1.53; 1.95)	1.78 (1.57; 2.01)	1.20 (1.12; 1.28)	1.08 (1.01; 1.16)
SUS-exclusive status	0.55 (0.5; 0.62)	0.53 (0.47; 0.59)	1.06 (0.99; 1.13)	1.10 (1.03; 1.18
Distance > 160 km residence to clinic	0.55 (0.44; 0.69)	0.57 (0.45; 0.72)	1.21 (1.06; 1.39)	1.18 (1.03; 1.35)
Time from first RA claim to first b/tsDMARD treatment—months	1.02 (1.01; 1.03)	1.01 (0.99; 1.02)	1.02 (1.02; 1.02)	1.01 (1.01; 1.01)
Number of prior bDMARD used	–	–	0.15 (0.14; 0.16)	0.15 (0.14; 0.16)
Number of prior csDMARD used	1.21 (1.11; 1.31)	1.23 (1.08; 1.41)	1.35 (1.31; 1.4)	1.16 (1.11; 1.22)

*DMARD* disease modifying antirheumatic drug, *JAKi* Janus kinase inhibitor, *b/tsDMARD* biologic/targeted synthetic/tofacitinib (JAKi) DMARD drug, *cDMARD* conventional DMARD, *CI* confidence interval, *N* number of patients, *ICD* International Classification of Disease, *SUS* Sistema Único de Saúde, S*US-exclusive* dependent on SUS for all healthcare resources, *SUS+ private* only dependent on SUS for prescription drug coverage.

Distance residence to clinic: distance was calculated as the Euclidean distance (km) between two postal codes: patient’s residence and the healthcare facility/institution. Cox ratio determines the switching pattern of bDMARD to JAKi and odds ratio determine the initiating rates of JAKi.

The logistic regression model showed that patients ≥ 50 years of age were 78% more likely to initiate a new oral tsDMARD. Patients residing more than 160 km from the health center were less likely to initiate treatment with tsDMARD (OR 0.55; p = 0.01). Neither gender nor time from first RA claim to first b/tsDMARDs were found to have a significant effect on RA treatment.

In the Cox multivariate models, patients older than 50 years of age were more likely to change from bDMARD to a new oral tsDMARD (HR 1.08; p = 0.03). Patients taking immunosuppressive csDMARD previously were 1.16 times more likely to take a novel oral tsDMARD (JAK inhibitor) (p = 0.005) in this study. Patients residing > 160 km of the pharmacy are 18% more likely to switch from a bDMARD to tsDMARD. There was no impact of gender on tsDMARD initiation.

### Usage of cs/imsDMARD drug prior to initiating b/tsDMARD

**Figure 3:**
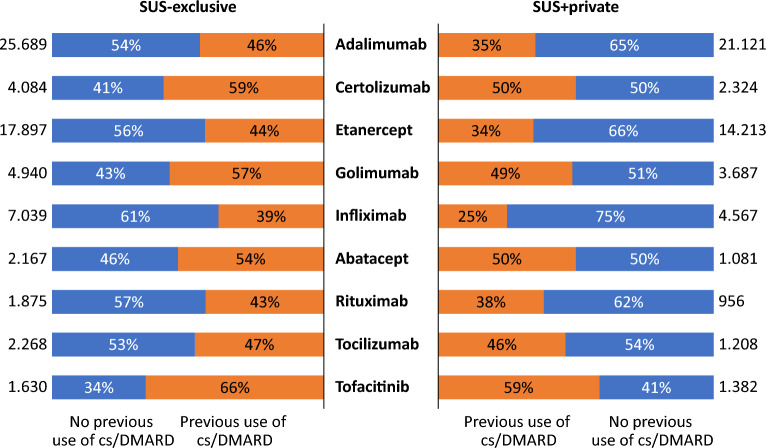
Previous use of csDMARD before initiating b/tsDMARD, stratified by SUS coverage dependency (exclusive or with private insurance) among patients with rheumatoid arthritis in DATASUS. *b/tsDMARD* biologics target synthetic disease modifying antirheumatic drug. tsDMARD limited to tofacitinib at time of analysis. SUS-exclusive, dependent on SUS for all healthcare resources; SUS+ private, individuals dependent on SUS only for prescription drug coverage. *csDMARD* conventional synthetic disease modifying antirheumatic.

The study assessed the use of bDMARDs or tofacitinib after the failure of conventional DMARDs (csDMARDs) (Fig. 3). For SUS-exclusive, only a marginal difference was evident in the proportion of patients undergoing adalimumab and etanercept treatments between populations who either had or lacked prior exposure to csDMARDs (46% vs. 54% for adalimumab and 44% vs. 56% for etanercept). However, most patients had previous exposure of csDMARDs in the case of certolizumab (59%) and golimumab (57%). For infliximab, however, the pattern was reversed: the majority (61%) lacked previous exposure to cs/imsDMARDs.

For the SUS+ private group, most patients undergoing adalimumab (65%), etanercept (66%) and infliximab (75%) treatment lacked prior exposure to csDMARDs, while it was comparable for certolizumab (50%) and golimumab (51%). Overall, there was less prior exposure to csDMARDs among SUS+ private patients.

### Treatment switching patterns of bDMARDs 

**Figure 4:**
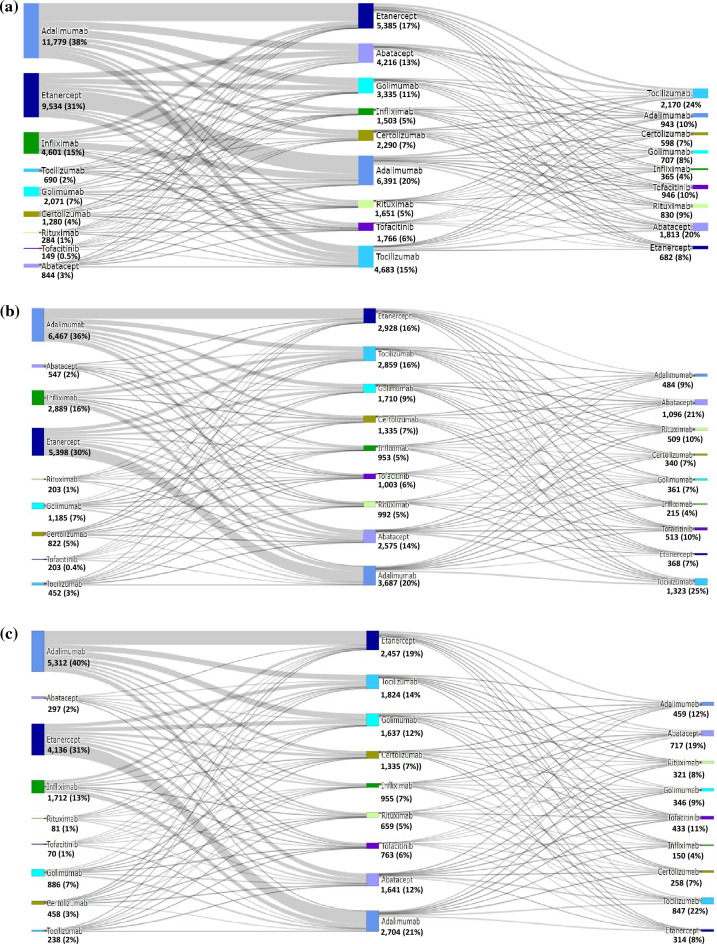
(**a**) Sankey diagram depicting treatment sequencing among patients with rheumatoid arthritis initiating a b/tsDMARD who transition to a second-line, and/or third-line treatment (N = 31,232). *b/tsDMARD* biologic target synthetic disease modifying antirheumatic drug; tsDMARD limited to tofacitiniv at time of analysis; Sankey diagram limited to b/tsDMARDs. csDMARDs, conventional disease modifying antirheumatics, not shown and may be combination with b/tsDMARDs). (**b**) Sankey diagram of patients with rheumatoid arthritics initiating a b/tsDMARD who transition to a second-line, or third-line treatment (SUS-exclusive cohort) in DATASUS (N = 18,042). *b/ts*DMARD biologic target synthetic disease modifying antirheumatic. tsDMARD limited to tofacitinib at time of analysis;Sankey limited to b/tsDMARDs (csDMARD, conventional disease modifying antirheumatics, not shown and may be combination with b/tsDMARDs); SUS-exclusive are individuals, dependent on SUS for all healthcare resources. (**c**) Sankey diagram of patients with rheumatoid arthritis initiating a b/tsDMARD who transition to a second-line and/or third-line treatment (SUS+ private insurance cohort)in DATASUS (N = 13,190). *b/tsDMARD* biologic target synthetic disease modifying antirheumatic. tsDMARD limited to tofacitinib at time of analysis. Sankey limited to b/tsDMARDs (csDMARDs, conventional disease modifying antirheumatics, not shown and may be combination with b/tsDMARDs). SUS+ private insurance are individuals dependent on SUS only for prescription drug coverage.

Sankey diagrams were developed to illustrate the number of patients that started first-line DMARDs at different periods of time for each drug (bDMARDs) and changes in treatment patterns (switching, discontinuation, or no change). Patients who did not switch were only represented in LOT1 (not contemplate in LOT2+). Patients on adalimumab treatment who transitioned to a different DMARD in LOT2 represented switching to etanercept (33.7%), tocilizumab (14.1%), and golimumab (13.7%), while those on etanercept changed to adalimumab (67%), abatacept (13.11%), and tocilizumab (12.8%) (Fig. 4a). Overall, the most common treatments in LOT3 were tocilizumab (2.17k) and abatacept (1.81k). Dynamic Sankey diagrams may be accessed online.

For the SUS-exclusive cohort (Fig. 4b), patients on LOT1 adalimumab were switched to LOT2 etanercept (31.9%), tocilizumab (14.4%), and abatacept (13.4%), whereas those on LOT1 etanercept were switched to LOT2 adalimumab (40.3%), abatacept (13.7%), and tocilizumab (13.5%).

For the SUS+ private cohort (Fig. 4c), patients on LOT1 adalimumab treatment changed to LOT2 etanercept (34.2%), tocilizumab (13.8%), and abatacept (12.2%), whereas those on LOT1 etanercept changed to LOT2 adalimumab (40.5%), golimumab (13.5%), and tocilizumab (11.6%).

### Time to first bDMARD therapy switch

**Figure 5:**
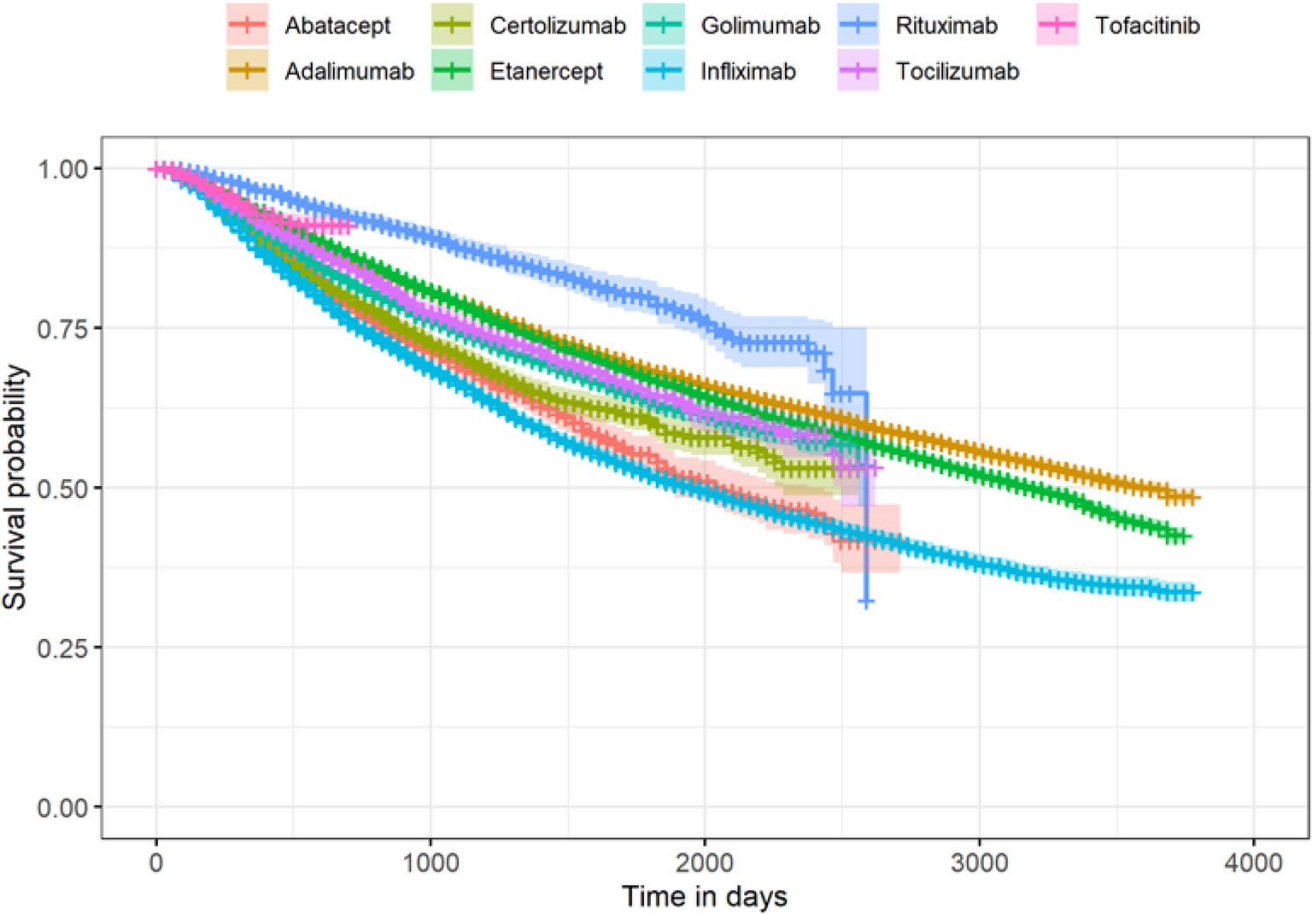
Kaplan Meier plot depicting timing of switch from first-line (LOT1) to second-line treatment (LOT2) among rheumatoid arthritis patients receiving b/tsDMARD therapies in DATASUS (2010–2020). *b/tsDMARD* biologic target synthetic disease modifying antirheumatic; *tsDMARD* limited to tofacitinib at the time of analysis.

Kaplan Meier analyses of time to switching from LOT1 to LOT2 are illustrated in Fig. 5. Adalimumab, etanercept, and infliximab were the bDMARDs that sustained longer length of time as a first-line therapy before a switch or censoring event. Adalimumab and etanercept had a comparable probability (48.6% for Adalimumab and 42.5% for Etanercept) of remaining on LOT1 by the end of the study period, whereas infliximab had a lower probability of 33.7%.

## Discussion

DATASUS is an administrative claims database and represents healthcare data from SUS, the largest government-run public health insurance system in the world by number of beneficiaries/users. From its inception, DATASUS was intended to enable researchers conduct health resource utilization and epidemiological studies relevant to the Brazilian population^13,27^. In the case of RA, to date, DATASUS has not been evaluated as a relevant and reliable data source for pharmacoepidemiologic research. Our study focused on DMARD therapies in RA among prevalent and new users, examining switching and discontinuation patterns over the last decade. We also evaluated determinants of initiation and/or switching to a novel therapy in this population.

In this study, we identified a large and representative sample of 250,251 individuals diagnosed with RA in DATASUS, treated with DMARDs between January 1st, 2010, and December 31st, 2020. The study cohort had a median age of 55 years, mostly female (82.8%), and white (57.7%). The characteristics of our cohort corroborated findings from earlier studies in Brazil^14,22,28,29^. Results from the REAL study (2020) of 1115 patients with RA treated at 11 public (SUS) centers across Brazil reported a median age of 56.6 years, 89.5% female, and 58.6% white^29^. Although potential genetic factors may contribute to RA predisposition among whites/Caucasians (proposed in REAL study)^29^, and more than half (52.9%) of patients in our study were from the south and southeast regions in Brazil where there is a high proportion of white/Western European ethnic population, this region benefits from better access to healthcare from a higher concentration of physicians^30,31^. The geographic heterogeneity found in our study is in line with previous Brazilian perspectives findings of patients with RA^32^.

In Brazil, pharmacoepidemiology research faces challenges, not from a lack of data, but rather from the lack of data integration and linked databases within SUS to ensure complete capture of the disease journey^33^. As with similar approaches outside of Brazil^9,17,18,20,22^, health record linkage methods were used to bridge data at the patient level across in-patient and out-patient data sets, thereby ensuring the longitudinal data capture across these settings to capture the full patient journey in RA^21,33^. Although, DATASUS has the advantage in capturing information from SUS comprehensive public health system, between 22 and 25% of the Brazilian population has supplementary health insurance^14,15,34^. This means that private insured patients may seek SUS to access high-cost medications while not seeking additional care within SUS. Because of this limitation in comprehensive data capture, we built a SUS+ private cohort and noted minor differences in treatment patterns in RA compared with the SUS-exclusive population, though no formal comparisons were performed.

The Brazilian Ministry of Health provides free access to nearly all currently approved synthetic and biological DMARDs for the treatment of RA within SUS, and new technologies are continuously assessed for possible incorporation^35^. The first anti-TNF agent to be introduced in SUS for RA was infliximab in 2002, followed by etanercept and adalimumab in 2006^35^. Currently, the Brazilian protocol for the management of RA does incorporate all relevant concepts and resources with proven efficacy in clinical trials^29^. There are concerns regarding extrapolating benefit-risk exclusively from clinical trial evidence to conditions in the real world, thus the critical role of identifying representative data sources that allow researchers to measure actual patterns of RA management in the real world.

In this study, we captured treatment patterns of newer and traditional biologic and targeted synthetic DMARDs that were introduced in Brazil over the last decade for treatment for RA. As expected per clinical guidelines and time on market in Brazil, adalimumab and etanercept were the most prevalent drugs used for RA treatment in the SUS-exclusive and SUS+ private cohorts over the study period. Similar patterns of etanercept and adalimumab dominance over other newly introduced agents have also been observed in other countries, such as the United States and Taiwan.^28,36,37^ Similarly, although tocilizumab and abatacept have emerged as newer DMARD treatments of choice in recent years, the follow-up time was limited compared to prevalent drugs. And, to address pharmacoepidemiologic questions centered on safety events of interest, which may be valid and deemed to be captured consistently in SUS, cycling between DMARDs and new drugs must be carefully considered over a longer length of follow-up time available for study. Because of SUS's comprehensive reimbursement for DMARDs, it is possible to analyze pattern shifts in treatment preference over time in parallel with physicians' experiences and clinical outcomes.

Switching among biologic therapies is considered when the first course of treatment demonstrates insufficient efficacy or adverse effects, although patient and physician preference may also play a role^38^. Furthermore, studies show that up to 40% of patients do not respond to available treatments, including DMARDs^39^. Based on previous observations, switching was considered an alternate approach for controlling disease progression and severity^40^. As anticipated based on approved indications^28^, we observed that patients treated with etanercept, adalimumab, and other index b/tsDMARDs in first line, switched to other b/tsDMARDs, mainly tocilizumab and abatacept for the second line RA treatment. Future patient and physician centered research may elicit details on reasons for drug treatment switching, which we could not address in DATASUS, an administrative claims database.

We evaluated patient characteristics found in DATASUS as potential predictors of initiating or switching to a tsDMARD, tofacitinib, a new therapeutic intervention representing a novel mechanism of action (JAKi) introduced in SUS in 2017 (approved for second or third line therapy in RA according to Clinical Guideline of Ministry of Health)^41^. Independent predictors of switching to and/or initiating tsDMARD included distance to clinic, number of previous biologic DMARDs, and being exclusively dependent on SUS for health insurance coverage. Individuals with RA who reside more than 160 km from a SUS healthcare center were 43% less likely to initiate tsDMARD and 18% more likely to switch from bDMARD to tsDMARD. Further studies are needed to effectively evaluate the influence of distance to clinic and treatment choice, but we hypothesize that, based on preference studies and appreciation of benefits associated with oral medications, physicians may be less likely to prescribe tsDMARD as first-line treatment for patients who live further away if they do not have an established relationship or have experience managing their disease using bDMARDs^42^. Conversely, the added convenience of an oral alternative may viewed as favorable or practical for patients who have been treated in the past with other bDMARDs.

Strengths related to the study included its large sample size, efficiency, generalizability, high validity, completeness of prescription drug information, and mapping of data for drug utilization research needs. Retrospective analyses of DATASUS make it possible to assess and compare many available biologics at the same time. A big advantage of DATASUS was the broad coverage in providing detailed information on patients with RA, as it encompasses 77.5% of the Brazilian population^14^.

Our study had limitations stemming from the inconsistent and incomplete data collection methods for DATASUS. In particular, limited clinical data, questionable accuracy of disease diagnosis, and the absence of cross-validated and linkage of healthcare data in Brazil are key challenges for studies of chronic diseases like RA^4,14^. Also, observational research studies may be subject to confounding and misclassification biases due to unmeasured and unknown variables associated with drug exposure. Disease activity is contributing factor that could not be evaluated using DATASUS. Smaller studies like the REAL study have longer follow up and capture disease activity and clinical response, which are not possible to ascertain from DATASUS claims^29^. Also, there may be an absence in consistency of data capture for information such as duration and doses of each medication as well as reasons for discontinuation. Future pharmacoepidemiology studies focused on safety objectives may need to focus on SUS-exclusive populations within DATASUS where health-related encounters, treatments, and procedures are consistently captured, granted that the outcomes of interest are measured and validated. Although multivariate regression analysis has been applied to control potential confounders, the independent effects of the treatment group on the decision of intra- or inter-biologic switching may be subject to omitted-variable biases, because some important information, such as laboratory data, disease duration, and disease activity, was not available in the dataset. Data fragmentation and accessibility problems in Brazil can be resolved systematic databases, further classified into SUS-exclusive and SUS+ private groups to optimize the ability to conduct pharmacoepidemiology research.

As with other health insurance administrative claims databases, DATASUS has the potential to be a valuable source of real-world data when handled with pharmacoepidemiologic principles of scientific study design and analyses. Often regarded as the foundation for pharmacoepidemiologic research, claims data are used for post-marketing drug surveillance, cost-effectiveness evaluations, and when evaluating preventive therapeutic strategies^10,20,43^. Our analyses of DATASUS provided insights on the treatment patterns relevant to individuals living with RA in Brazil that may contribute to a more accurate prediction of future healthcare planning, helping to adapt the Brazilian healthcare system to these changes.

## Conclusion

The present population-based study in Brazil investigated treatment patterns of newer and traditional biologic and targeted synthetic DMARDs that were introduced in Brazil over the last decade for treatment for RA. The advantage of DATASUS was its broad coverage of approximately 77.5% of the Brazilian population, making it a valuable and representative data source for treatment-related analyses for pharmacoepidemiology and healthcare resource utilization research of the public health system in Brazil. In the case of RA, although disease severity and lab values were not obtainable from these administrative claims data, we identified potential factors such as age, distance from clinic, and previous exposure to DMARDs associated with switching or initiating a novel DMARD (JAKi). These findings may help to support and plan resource allocation, rational use of drugs and financial resources in Brazil and will further aid in designing health policy guidelines.

### Supplementary Information

Supplementary Table S1.

Supplementary Information 1.

Supplementary Information 2.

Supplementary Information 3.

## Data Availability

The data generated during and/or analyzed during the current study are available on DATASUS website. DATASUS is a database established by the Brazilian Ministry of Health that contains information and statistics from all municipalities in Brazil and is publicly available through online access (http://datasus.saude.gov.br/).

## References

[1] Corrigan-Curay J, Sacks L, Woodcock J (2018). Real-world evidence and real-world data for evaluating drug safety and effectiveness. JAMA.

[2] Katkade VB, Sanders KN, Zou KH (2018). Real world data: An opportunity to supplement existing evidence for the use of long-established medicines in health care decision making. J. Multidiscip. Healthc..

[3] U.S. Food and Drug. *Real-World Evidence*. https://www.fda.gov/science-research/science-and-research-special-topics/real-world-evidence (2023).

[4] Leal LF (2022). Data sources for drug utilization research in Brazil—DUR-BRA Study. Front. Pharmacol..

[5] Ogale S, Hitraya E, Henk HJ (2011). Patterns of biologic agent utilization among patients with rheumatoid arthritis: A retrospective cohort study. BMC Musculoskelet. Disord..

[6] Fakhouri W (2018). Treatment patterns, health care resource utilization and costs of rheumatoid arthritis patients in Italy: Findings from a retrospective administrative database analysis. Open Access Rheumatol..

[7] Methodology, W. H. O. I. W. G., Methodology, W. H. O. C. C., Research, W. H. O. C. C. & Clinical Pharmacological. *Introduction to Drug Utilization Research*. (2003).

[8] Lopes LC (2022). Data sources for drug utilization research in Latin American countries—A cross-national study: DASDUR-LATAM study. Pharmacoepidemiol. Drug Saf..

[9] Justo N (2019). Real-World evidence in healthcare decision making: Global trends and case studies from Latin America. Value Health.

[10] Dutcher, S., Maro, J. C. & Martin, D. *Medical Product Safety: Ten Years of the U.S. Sentinel System*. https://www.sentinelinitiative.org/news-events/publications-presentations/medical-product-safety-ten-years-us-sentinel-system (2019).

[11] Brasil, Ministério da Saúde, Secretaria de Atenção à Saúde. *Manual Técnico Operacional SIA/SUS Sistema de Informações Ambulatoriais*. Vol. 69. (Ministério da Saúde, 2010).

[12] Machado JP, Martins M, da Leite IC (2016). Qualidade das bases de dados hospitalares no Brasil: Alguns elementos. Rev. Bras. Epidemiol..

[13] Ministério da Saúde. *Agência Nacional de Saúde Suplementar*. https://www.gov.br/ans/pt-br.

[14] da Rocha Castelar-Pinheiro G (2018). The REAL study: A nationwide prospective study of rheumatoid arthritis in Brazil. Adv. Rheumatol..

[15] Montekio VB, Medina G, Aquino R (2011). The health system of Brazil. Salud Publ. Mex..

[16] Almutairi K, Nossent J, Preen D, Keen H, Inderjeeth C (2021). The global prevalence of rheumatoid arthritis: A meta-analysis based on a systematic review. Rheumatol. Int..

[17] Gomes RM (2016). Ten-year kidney transplant survival of cyclosporine- or tacrolimus-treated patients in Brazil. Expert Rev. Clin. Pharmacol..

[18] Smolen JS (2017). EULAR recommendations for the management of rheumatoid arthritis with synthetic and biological disease-modifying antirheumatic drugs: 2016 update. Ann. Rheum. Dis..

[19] Julian GS, Rosim RP, Carneseca EC, Rigolon J (2020). Annualized hospitalization rate with natalizumab vs fingolimod in second-line treatment for RRMS in the public healthcare system in Brazil: A claim database approach. PLoS One.

[20] Barbour J (2021). Healthcare resource utilization of spinal muscular atrophy in the Brazilian Unified Health System: A retrospective database study. J. Bras. Econ. Saúde.

[21] Sanni Ali M (2019). Administrative data linkage in Brazil: Potentials for health technology assessment. Front. Pharmacol..

[22] Touma-Falci L (2021). Age-related macular degeneration and resource utilization in the Brazilian public healthcare system: A real-world retrospective study. BMC Ophthalmol..

[23] Brasil, Ministério da Saúde, Datasus. *SIGTAP—Sistema de Gerenciamento da Tabela de Procedimentos, Medicamentos e OPM do SUS*. https://websaude.org/sigtap-tabela-de-procedimentos-medicamentos-orteses-proteses-e-materiais-especiais/#:~:text=O%20Sistema%20de%20Gerenciamento%20da%20Tabela%20de%20Procedimentos%2C,os%20atributos%20de%20cada%20procedimento%2C%20compatibilidades%20e%20relacionamentos (2018).

[24] Freire SM, De Souza RC, de Almeida RT (2015). Integrating brazilian health information systems in order to support the building of data warehouses. Rev. Bras. Eng. Biomed..

[25] Campos, D., Rosim, R., Duva, A. & Ballalai Ferraz, A. *Brazilian Healthcare Record Linkage (BRHC-RLK)–A Record Linkage Methodology for Brazilian Medical Claims Datasets (DATASUS)*. *Value Health*. In *22nd International Abstracts Book*. Vol. 20. A1–A383 (2017).

[26] Diniz IM (2018). The long-term costs for treating multiple sclerosis in a 16-year retrospective cohort study in Brazil. PLoS One..

[27] The World Bank-Brazil. *Population, Total-Brazil*. https://data.worldbank.org/indicator/SP.POP.TOTL?locations=BR.

[28] Li KJ, Chang CL, Hsin CY, Tang CH (2021). Switching and discontinuation pattern of biologic disease-modifying antirheumatic drugs and tofacitinib for patients with rheumatoid arthritis in Taiwan. Front. Pharmacol..

[29] Gomides APM (2020). Rheumatoid artrhitis treatment in Brazil: Data from a large real-life multicenter study. Adv. Rheumatol..

[30] Gandelman Horovitz DD, De Faria Ferraz VE, Dain S, Marques-De-Faria AP (2013). Genetic services and testing in Brazil. J Commun. Genet..

[31] CatussiPaschoalotto MA, Passador JL, Passador CS, Endo GY (2022). Regionalization of health services in Brazil: An analysis of socioeconomic and health performance inequalities. Gestão Region..

[32] Cavalcanti FS (2010). Management of rheumatoid diseases: The Brazilian perspective. Rheumatology.

[33] Guerra AA (2018). Building the national database of health centred on the individual: Administrative and epidemiological record linkage—Brazil, 2000–2015. Int. J. Popul. Data Sci..

[34] Ministério da Saúde—Anvisa. *Sistema Nacional de Gerenciamento de Produtos Controlados*. http://portal.anvisa.gov.br/produtos_controlados (2019).

[35] de Ávila Machado MA (2016). Treatment persistence in patients with rheumatoid arthritis and ankylosing spondylitis. Rev. Saude Publ..

[36] Desai RJ, Solomon DH, Jin Y, Liu J, Kim SC (2017). Temporal trends in use of biologic DMARDs for rheumatoid arthritis in the United States: A cohort study of publicly and privately insured patients. J. Manag. Care Spec. Pharm..

[37] Silva BS, Coelho HV, Cavalcante RB, de Oliveira VC, de Guimarães EAA (2018). Evaluation study of the national immunization program information system. Rev. Bras. Enferm..

[38] Cannon GW (2016). Clinical outcomes and biologic costs of switching between tumor necrosis factor inhibitors in US veterans with rheumatoid arthritis. Adv. Ther..

[39] Bonfiglioli KR (2021). Recommendations of the Brazilian Society of Rheumatology for the use of JAK inhibitors in the management of rheumatoid arthritis. Adv. Rheumatol..

[40] Favalli EG, Biggioggero M, Marchesoni A, Meroni PL (2014). Survival on treatment with second-line biologic therapy: A cohort study comparing cycling and swap strategies. Rheumatology..

[41] PCDT. *Clinical Protocol and Therapeutic Guidelines for Rheumatoid Arthritis*. (2020).

[42] van Heuckelum M (2019). Preferences of patients with rheumatoid arthritis regarding disease-modifying antirheumatic drugs: A discrete choice experiment. Patient Prefer. Adherence.

[43] Areco KN (2021). Operational challenges in the use of structured secondary data for health research. Front. Public Health.

