# Supplementary material for: Real-world treatment patterns of rheumatoid arthritis in Brazil: analysis of DATASUS national administrative claims data for pharmacoepidemiology studies (2010–2020)
Source: Sci Rep. 2023 Oct 18;13:17739. doi: 10.1038/s41598-023-44389-9 (PMC10584810; doi:10.1038/s41598-023-44389-9)
Supplement: Supplementary file 1 — Supplementary Table S1. [file 41598_2023_44389_MOESM1_ESM.pdf]

**Supplementary**

Supplementary Table 1. RA treatment categories and definitions

| DMARD type | Drug class | Drug* | Codes (SIGTAP) |
|---|---|---|---|
| Cs/DMARDs | csDMARD | Ciclosporin | 0604340010, 0604340044, 0604340052, 0603020020, 0603020038, 0604340028, 0603020046, 0604340036, 0603020054, 0603020011, 0601200020, 0601200039, 0601200047, 0601200055, 0601200063 |
| | | Cyclophosphamide | 0604090013, 0601200187 |
| | | Chloroquine | 0604080018, 0601080017 |
| | | Hydroxychloroquine | 0604080226, 0601080025 |
| | | Leflunomide | 0604320043, 0601200071 |
| | | Methotrexate | 0604530021, 0604530030, 0601200080, 0601200098 |
| | Immunosuppressant | Azathioprine | 0604530013, 0601200012 |
| | | Sulfasalazine | 0604010095, 0601090098 |
| bDMARDs | Other bDMARD | Abatacept | 0604320140, 0604320124 |
| | | Rituximab | 0604680023, 0604680331 |
| | | Tocilizumab | 0604690010 |
| | TNFi | Adalimumab | 0604380011, 0604380097, 0601010019 |
| | | Certolizumab | 0604380070 |
| | | Etanercept | 0604380020, 0604380038, 0604380100, 0601010027, 0601010051 |
| | | Golimumab | 0604380089 |
| | | Infliximab | 0604380046, 0601010035 |
| tsDMARDs | JAK inhibitor | Tofacitinib | 0604320159 |
